# N-Substituted Pyrido-1,4-Oxazin-3-Ones Induce Apoptosis of Hepatocellular Carcinoma Cells by Targeting NF-κB Signaling Pathway

**DOI:** 10.3389/fphar.2018.01125

**Published:** 2018-11-05

**Authors:** Chakrabhavi Dhananjaya Mohan, Hanumantharayappa Bharathkumar, Dukanya, Shobith Rangappa, Muthu K. Shanmugam, Arunachalam Chinnathambi, Sulaiman Ali Alharbi, Tahani Awad Alahmadi, Atanu Bhattacharjee, Peter E. Lobie, Amudha Deivasigamani, Kam Man Hui, Gautam Sethi, Basappa, Kanchugarakoppal S. Rangappa, Alan Prem Kumar

**Affiliations:** ^1^Department of Studies in Molecular Biology, University of Mysore, Mysore, India; ^2^Laboratory of Chemical Biology, Department of Chemistry, Bangalore University, Bangalore, India; ^3^Department of Studies in Organic Chemistry, University of Mysore, Mysore, India; ^4^Adichunchanagiri Institute for Molecular Medicine, Mandya, India; ^5^Department of Pharmacology, Yong Loo Lin School of Medicine, National University of Singapore, Singapore, Singapore; ^6^Department of Botany and Microbiology, College of Science, King Saud University, Riyadh, Saudi Arabia; ^7^Department of Pediatrics, College of Medicine and King Khalid University Hospital, King Saud University Medical City, Riyadh, Saudi Arabia; ^8^Department of Biotechnology & Bioinformatics, North Eastern Hill University, Shillong, India; ^9^Tsinghua Berkeley Shenzhen Institute and Division of Life Science and Health, Tsinghua University Graduate School, Shenzhen, China; ^10^Division of Cellular and Molecular Research, Humphrey Oei Institute of Cancer Research, National Cancer Centre, Singapore, Singapore; ^11^Institution of Excellence, Vijnana Bhavan, University of Mysore, Mysore, India; ^12^Cancer Science Institute of Singapore, National University of Singapore, Singapore, Singapore; ^13^Cancer Program, Medical Science Cluster, Yong Loo Lin School of Medicine, National University of Singapore, Singapore, Singapore; ^14^Curtin Medical School, Faculty of Health Sciences, Curtin University, Perth, WA, Australia

**Keywords:** oxazines, anticancer, NF-κB, hepatocellular carcinoma, apoptosis

## Abstract

Hepatocellular carcinoma (HCC) is a fatal disease and ranked fifth in cancer related mortality. Persistent activation of NF-κB is responsible for the oncogenesis, metastasis, tumor evasion, anti-apoptosis, angiogenesis and proliferation in HCC. Therefore, designing of chemically novel, biologically potent small molecules that target NF-κB signaling cascade have gained prominent clinical interest. Herein we synthesized a novel class of 4-(substituted)-2H-pyrido[3,2-b][1,4]oxazin-3(4H)-one by reacting 2H-pyrido[3,2-b][1,4]oxazin-3(4H)-one with various alkyl halides by using combustion derived bismuth oxide. We evaluated the antiproliferative efficacy of newly synthesized compounds against HCC cells and identified 4-(4-nitrobenzyl)-2H-pyrido[3,2-b][1,4]oxazin-3(4H)-one (NPO) as lead anticancer agent. In addition, we investigated the effect of NPO on the DNA binding ability of NF-κB and NF-κB regulated luciferase expression in HCC cells. The results demonstrated that NPO can induce significant growth inhibitory effects in HepG2, HCCLM3 and Huh-7 cells in dose and time-dependent manner. Interestingly, NPO induced significant downregulation in p65 DNA binding ability, p65 phosphorylation and subsequent expression of NF-κB dependent luciferase gene expression in diverse HCC cell lines. Further, *in silico* docking analysis suggested that NPO can show direct physical interaction with NF-κB. Finally, NPO was found to significantly abrogate tumor growth at a dose of 50 mg/kg in an orthotopic mouse model. Thus, we report the potential anticancer effects of NPO as a novel inhibitor of NF-κB signaling pathway in HCC.

## Introduction

Hepatocellular carcinoma (HCC) is one of the leading common cancers threatening the globe with high mortality rate (Subramaniam et al., 2013; Bharathkumar et al., 2014; Dai et al., 2015; Swamy et al., 2017). HCC ranks fifth most common cancer in men and ninth in women with high mortality rate all over the world (Flores and Marrero, 2014; Ashtari et al., 2015; Ferlay et al., 2015; Dai et al., 2016). Alcoholic hepatitis, non-alcoholic steatohepatitis, endemic hepatitis B or C viral infections, hemochromatosis, obesity, and consumption of aflatoxin B1 remains as the major factors contributing to the development of HCC (Subramaniam et al., 2013; Mohan et al., 2014). Moreover, HCC is often diagnosed at advanced stages in which the currently available therapeutic agents exhibit limited efficacy and contribute to poor prognosis (Chen et al., 2015). However, early detection and development of new therapeutics can contribute to improved prognosis in HCC patients.

Nuclear factor kappa B (NF-κB) is a latent pro-inflammatory transcription factor identified by Baltimore and colleagues in 1986 and distributed enormously in the cytoplasm of most of the mammalian cells (Ahn et al., 2007; Sethi et al., 2007, 2012; Shin et al., 2014; Neelgundmath et al., 2015; Puar et al., 2018). Depending on the cell type and cellular environment, various ligands including TNF-α, IL-1β, LTβ, LPS, CD40L, and BAFF relay signal for the activation of NF-κB (Sethi et al., 2006; Dey et al., 2008; Li et al., 2015b). In the absence of activation by various ligands, NF-κB remains associated with inhibitory-κB (IκB) in the cytoplasm (Li and Sethi, 2010; Manu et al., 2011; Chai et al., 2015; Puar et al., 2018). The interaction of specific ligand with its receptor triggers the activation of upstream kinases that can lead to the phosphorylation, ubiquitination and degradation of IκB and thereby activation of NF-κB which can translocate into nucleus (Sethi and Tergaonkar, 2009; Samy et al., 2012; Keerthy et al., 2014; Manu et al., 2014; Mohan et al., 2018). NF-κB also regulates the expression of nearly five hundred genes which are essential for the maintenance of homeostatic condition and promotion of oncogenesis (Gupta et al., 2010; Li et al., 2013, 2015a; Manu et al., 2015; Ningegowda et al., 2017). Persistent activation of NF-κB has been reported in several pro-inflammatory diseases including cancers, ulcerative colitis, Crohn’s disease, asthma and rheumatoid arthritis (Yamamoto and Gaynor, 2001; Aggarwal and Harikumar, 2009; Manu et al., 2012). Thus, designing novel NF-κB inhibitors may provide a promising therapeutic approach for the treatment of HCC.

The anti-tumor potential of oxazine-based compounds have been studied extensively in several cancer models and they have been reported to exhibit significant activity both *in vitro* and *in vivo* (Basappa Murugan et al., 2010; Bharathkumar et al., 2015). We have previously reported the *in vitro* NF-κB inhibitory activity of novel oxazines in colon cancer cells and *in vivo* inflammatory bowel disease model (Nirvanappa et al., 2016). In another study, 2-ethoxy-4,5-diphenyl-1,3-oxazine-6-one was reported to downregulate nuclear NF-κB in NGF-differentiated PC12 cells induced with lipopolysaccharide (Ansari et al., 2011). In addition, oxazine-tocotrienol conjugates have been demonstrated to decrease phosphorylation of NF-κB and IκB in + SA mammary tumor growth in syngeneic mice (Ananthula et al., 2014). Furthermore, it has been reported that derivatives of 1,3-oxazine and 1,3-benzoxazine show potent anticancer activity against lung and colon cancer cells. 3,5-Bis(2-pyridinylmethylidene)-4-piperidone, a pyridine derivative was reported to act as a potent inhibitor of LPS-induced NF-κB DNA binding activity (Olivera et al., 2012). Overall, these studies suggest that oxazines and pyridine-based small molecules could serve as important therapeutic agents to target various malignancies. Therefore, in continuation of our efforts to synthesize and explore medicinal properties of various heterocyclic compounds (Rangappa and Basappa, 2005; Srinivas et al., 2015; Anusha et al., 2016; Mohan et al., 2016, 2018; Sebastian et al., 2016), in the present article, we have reported the synthesis of pyrido-1,4-oxazin-3-ones and have also examined their anticancer potential against panel of HCC cells. Additionally, we have identified 4-(4-nitrobenzyl)-2H-pyrido[3,2-b][1,4]oxazin-3(4H)-one (5e, NPO) can function as potent anticancer agent and demonstrated its potential effects on cell viability, apoptosis, NF-κB activation in HCC cells and tumor growth in preclinical mouse model.

## Materials and Methods

### Chemistry

All chemicals used were of analytical grade and purchased from Sigma Aldrich, and SRL, Mumbai (India). ^1^H NMR spectra were recorded on a Agilent (400 MHz) spectrometer in CDCl_3_ solvent, using TMS as an internal standard, ^13^C NMR spectra were recorded on a Agilent (100 MHz) spectrometer and chemical shifts were expressed as δ ppm and abbreviations are assigned as, s = singlet, d = doublet, t = triplet, q = quartet, m = multiplet and J values are given in Hz. Mass spectra were determined on a Shimadzu LC-MS, elemental analyses were carried out using an Elemental Vario Cube CHNS Rapid Analyzer. Progress of the reaction was monitored by TLC pre-coated silica gel plates.

### General Procedure for the Synthesis of N-Substituted Pyrido-1,4-Oxazin-3-Ones

N-substituted pyrido-1,4-oxazin-3-ones were prepared in two step reaction. Initially, 2H-pyrido[3,2-b][1,4]oxazin-3(4H)-one was prepared as described earlier (Ankalgi and Ranawat, 2012). In brief, 2-aminopyridine-3-ol (**1**) was made to react with chloroacetylchloride (**2**) at 5°C in basic medium to generate 2H-pyrido[3,2-b][1,4]oxazin-3(4H)-one (**3**). In the next step, mixture of 2H-pyrido[3,2-b][1,4]oxazin-3(4H)-one(**3**), benzyl halide(**4**) and combustion derived bismuth oxide (1.5 mmol) was taken in a 50 ml round bottom flask and stirred for 5 h in DMF solvent at 40°C. Progress of the reaction was monitored by TLC. After completion of the reaction, catalyst was filtered followed by the addition of water to the filtrate. Thereafter, final compound is extracted using ethyl acetate, washed with brine solution, dried with anhydrous sodium sulfate and concentrated using rotary evaporator. The title compounds are purified by column chromatography using ethyl acetate and hexane as eluent. Combustion derived Bi_2_O_3_was prepared using previously reported protocol from our laboratory (Anusha et al., 2014).

### 4′-((3-Oxo-2H-Pyrido[3,2-b][1,4]Oxazin-4(3H)-yl)Methyl)-[1,1′-Biphenyl]-2-Carbonitrile (5a)

Compound **5a** was prepared from pyrido1,4-oxazin-3-one **3** (1mmol), 4′-(bromomethyl)-[1,1′-biphenyl]-2-carbonitrile **4a** (1.2 mmol) and combustion derived bismuth oxide, yield 97%, melting point 134–136°C, Elemental analysis calculated for C_21_H_15_N_3_O_2_: C, 73.89; H, 4.43; N, 12.31; found C, 73.64; H, 4.51; N, 12.23%; ^1^H NMR (CDCl_3_, 400 MHz) δ:8.04-8.02(d, J = 1.2 Hz, 1H), 7.74-7.71(m,1H), 7.60-7.56(m, 3H), 7.48-7.40(m, 4H), 7.24-7.22(m, 1H), 6.95-6.92(m,1H), 5.40(s, 2H), 4.72(s, 2H); ^13^C NMR(CDCl_3_, 100 MHz):164.61, 145.12, 141.42, 141.09, 140.67, 137.70, 137.13, 133.75, 132.75, 130.03, 129.40, 129.18, 128.97, 128.75, 127.45, 123.55, 119.39, 118.67, 111.13, 67.44, 42.37.Mass:m/z found for C_21_H_15_N_3_O_2_ was 342.2 (M + 1)^+^.

### 4-((6,6-Dimethyl-4-Phenyl-5,6-Dihydro-4H-1,2-Oxazin-3-yl)Methyl)-2H-Pyrido[3,2-b][1,4]Oxazin-3(4H)-One (5b)

Compound **5b** was prepared from pyrido1,4-oxazin-3-one **3** (1 mmol), 3-(bromomethyl)-6,6-dimethyl-4-phenyl-5,6-dihydro-4H-1,2-oxazine **4b** (1.2 mmol) and combustion derived bismuth oxide, yield 94%, melting point 67–68°C, Elemental analysis calculated for C_20_H_21_N_3_O_3_: C, 68.36; H, 6.02; N, 11.96; found C, 68.29; H, 6.09; N, 12.02%; ^1^H NMR(CDCl_3_, 400 MHz) δ: 7.95(d, 1H), 7.4-7.3(m, 5H), 7.2(d,1H), 6.9(t, 1H), 4.6(s, 2H), 4.4(s, 2H), 3.6(t,1H), 2.1-1.8(m, 2H), 1.3(s, 6H). Mass: m/z found forC_20_H_21_N_3_O_3_ was 352.22(M + 1)^+^.

### 4-(4-Isopropylbenzyl)-2H-Pyrido[3,2-b][1,4]Oxazin-3(4H)-One (5c)

Compound **5c** was prepared from pyrido1,4-oxazin-3-one **3** (1 mmol),1-(bromomethyl)-4-isopropylbenzene **4c** (1.2 mmol) and combustion derived bismuth oxide, yield 93%, melting point 78–79°C, Elemental Analysis Calculated for C_17_H_18_N_2_O_2_: C, 72.32; H, 6.43; N, 9.92; found C, 72.25; H, 6.30; N, 10.02%; ^1^H NMR(CDCl_3_, 400 MHz) δ:8.02-8.01 (d, J = 4.8Hz, 1H,Ar-H), 7.39-7.37(m,2H, Ar-H), 7.20-7.12(m,3H), 6.92-6.89(m,1H), 5.31(s, 2H), 4.68(s, 2H), 2.86-2.82(m,1H), 1.20-1.18(d,6H); ^13^C NMR(CDCl_3_, 100 MHz):164.51, 147.89, 141.62, 141.03, 140.68, 134.45, 128.64, 127.41, 126.57, 126.36, 123.31, 119.15, 67.42, 42.35, 33.83, 23.95, 23.89.Mass:m/z found for C_17_H_18_N_2_O_2_ was 283.18 (M + 1)^+^.

### 4-(4-(Tert-Butyl)Benzyl)-2H-Pyrido[3,2-b][1,4]Oxazin-3(4H)-One (5d)

The compound **5d** was prepared from pyrido1,4-oxazin-3-one **3** (1mmol),1-(bromomethyl)-4-(tert-butyl)benzene **4d** (1.2 mmol) and combustion derived bismuth oxide, yield 94%, melting point 75–76°C, Elemental analysis calculated for C_18_H_20_N_2_O_2_: C, 72.95; H, 6.80; N, 9.45; found C, 72.86; H, 6.71; N, 9.37%;^1^H NMR(CDCl_3_, 400 MHz) δ:8.03-8.01(d, J = 1.6 Hz, 1H), 7.40-7.38(m,2H), 7.30-7.28(m, 2H), 7.21-7.18(m,1H), 6.92-6.89(m, 1H), 5.32(s, 2H), 4.68(s, 2H), 1.26(s, 9H); ^13^C NMR (CDCl_3_, 100 MHz):164.52, 150.15, 141.63, 141.03, 140.67, 134.07, 128.35, 126.87, 126.15, 125.42, 123.33, 119.16, 67.42, 42.26, 34.42, 31.30, 31.29, 31.14; Mass:m/z found forC_18_H_20_N_2_O_2_was 297.20(M + 1)^+^.

### 4-(4-Nitrobenzyl)-2H-Pyrido[3,2-b][1,4]Oxazin-3(4H)-One (5e)

Compound **5e** was prepared from pyrido1,4-oxazin-3-one **3** (1mmol),1-(bromomethyl)-4-nitrobenzene **4e** (1.2 mmol) and combustion derived bismuth oxide, yield 96%, melting point 68–69°C, Elemental analysis calculated for C_14_H_11_N_3_O_4_: C, 58.95; H, 3.89; N, 14.73; found C, 58.82; H, 3.77; N, 14.61%; ^1^H NMR(CDCl_3_, 400 MHz) δ:8.14-8.12(m, 2H), 7.99-7.98(d, J = 1.6, 1H), 7.60-7.58(m, 2H), 7.26-7.23(m, 1H), 6.97-6.94(m,1H), 5.41(s, 2H), 4.72(s, 2H);^13^C NMR(CDCl_3_, 100 MHz); 164.63, 147.26, 144.42, 141.15, 141.05, 140.62, 129.29, 129.96, 123.73, 123.66, 123.59, 119.69, 67.38, 42.20;Mass: m/z found for C_14_H_11_N_3_O_4_ 286.12 (M + 1)^+^.

### 4-((4-(4-Chlorophenyl)-6,6-Dimethyl-5,6-Dihydro-4H-1,2-Oxazin-3-yl) Methyl)-2H-Pyrido[3,2-b][1,4]Oxazin-3(4H)-One (5f)

Compound **5f** was prepared from pyrido1,4-oxazin-3-one **3** (1 mmol),3-(bromomethyl)-4-(4-chlorophenyl)-6,6-dimethyl-5,6-dihydro-4H-1,2-oxazine **4f** (1.2 mmol) and combustion derived bismuth oxide, yield 95%, melting point 89–90°C, Elemental analysis calculated for C_20_H_20_ClN_3_O_3_: C, 62.26; H, 5.22; N, 10.89; found C, 62.19; H, 5.15; N, 10.79%;^1^H NMR(CDCl_3_, 400 MHz) δ: 7.95(d, 1H), 7.4-7.2(m, 5H), 6.9(t, 1H), 4.6(s, 2H), 4.45(s, 2H), 3.6(t, 1H), 2.1-1.7(m, 2H), 1.3(s, 6H).; Mass: m/z found forC_20_H_20_ClN_3_O_3_ was 386.17 (M + 1)^+^.

### 4-((3-Oxo-2H-Pyrido[3,2-b][1,4]Oxazin-4(3H)-yl)Methyl)Benzonitrile (5g)

Compound **5g** was prepared from pyrido1,4-oxazin-3-one **3** (1 mmol),4-(bromomethyl)benzonitrile **4g** (1.2 mmol) and combustion derived bismuth oxide, yield 91%, melting point 59–60°C, Elemental analysis calculated for C_15_H_11_N_3_O_2_: C, 67.92; H, 4.18; N, 15.84; found C, 67.85; H, 4.10; N, 15.75%; ^1^H NMR (CDCl_3_, 400 MHz) δ:8.0 (d, 1H), 7.7-7.6(m, 4H), 7.24(m, 1H), 7.0(d, 1H), 5.4 (s, 2H), 4.7(s, 2H); Mass: m/z found forC_15_H_11_N_3_O_2_was 266.12 (M + 1)^+^.

### 4-(Cyclohexylmethyl)-2H-Pyrido[3,2-b][1,4]Oxazin-3(4H)-One (5h)

Compound **5h** was prepared from pyrido1,4-oxazin-3-one **3** (1mmol),(bromomethyl)cyclohexane **4h** (1.2 mmol) and combustion derived bismuth oxide, yield 93%, melting point 60–62°C, Elemental Analysis Calculated for C_14_H_18_N_2_O_2_: C, 68.27; H, 7.37; N, 11.37; found C, 68.18; H, 7.29; N, 11.31%;^1^H NMR(CDCl_3_, 400 MHz) δ:8.00-7.99(d, J = 1.6Hz,1H), 7.21-7.19(d, J = 1.6Hz, 1H), 6.92-6.89(m, 1H), 4.64(s, 2H), 4.02-4.00(d, J = 7.2 Hz, 2H), 1.85-1.82(m, 1H), 1.69-1.57(m, 5H), 1.18-1.14(m, 3H), 1.07-1.01(m, 2H); Mass: m/z found for C_14_H_18_N_2_O_2_was 247.0(M + 1)^+^.

### 2-(3-(3-Oxo-2H-Pyrido[3,2-b][1,4]Oxazin-4(3H)-yl)Propyl)Isoindoline-1,3-Dione (5i)

Compound **5i** was prepared from pyrido1,4-oxazin-3-one **3** (1 mmol),2-(3-bromopropyl)isoindoline-1,3-dione **4i** (1.2 mmol) and combustion derived bismuth oxide, yield 95%, melting point 142–143°C, Elemental Analysis Calculated for C_18_H_15_N_3_O_4_: C, 64.09; H, 4.48; N, 12.46; found C, 64.01; H, 4.40; N, 12.38%;^1^H NMR(CDCl_3_, 400 MHz) δ: 7.9-7.8(m,3H), 7.8-7.7(m, 2H), 7.2(d, 1H), 6.9(t, 1H), 4.7(s, 2H), 4.2(t, 2H), 3.8(t, 2H), 2.2(m, 2H); ^13^C NMR (CDCl_3_, 100 MHz):168.26, 164.55, 141.41, 140.96, 140.59, 133.99, 133.79, 132.15, 123.26, 123.11, 119.08, 67.29, 47.24, 36.70, 26.83; Mass: m/z found for C_18_H_15_N_3_O_4_ was 338.1(M + 1)^+^.

### 4-(2,6-Dichlorobenzyl)-2H-Pyrido[3,2-b][1,4]Oxazin-3(4H)-One(5j)

Compound **5j** was prepared from pyrido1,4-oxazin-3-one **3** (1 mmol),2-(bromomethyl)-1,3-dichlorobenzene **4j** (1.2 mmol) and combustion derived bismuth oxide, yield 94%, melting point 100–102°C, Elemental Analysis Calculated for C_14_H_10_Cl_2_N_2_O_2_: C, 54.39; H, 3.26; N, 9.06; found C, 54.30; H, 3.31; N, 9.01%;^1^H NMR(CDCl_3_, 400 MHz) δ:8.0(d, 1H), 7.3-7.2(m, 3H), 7.1(d, 1H), 6.9(d, 1H), 5.6(s, 2H), 4.6 (s, 2H); Mass: m/z found for C_14_H_10_Cl_2_N_2_O_2_ was 309.0(M + 1)^+^310.0(M + 2)^+^.

## Pharmacology

### Cell Lines

HepG2 cell line was obtained from American Type Culture Collection. Huh-7 cell line was obtained from Japanese Collection of Research Bioresources (JCRB) Cell Bank (Osaka, Japan) and HCCLM3 cell line was a kind gift from Professor Zhao-You Tang at the Liver Cancer Institute (Zhongshan Hospital, Fudan University, Shanghai, China). All the HCC cells were cultured in Dulbecco’s Modified Eagle Medium (DMEM) with 10% FBS.

### MTT Assay

The growth inhibitory activity of newly synthesized pyrido [1,4]oxazinones against HCC cells was determined by the MTT dye uptake method as described earlier (Baburajeev et al., 2015). Briefly, HCC cells (2.5 × 10^4^/ml) were incubated in triplicate in a 96-well plate, in the presence of varying compound concentrations at a volume of 0.2 ml, for different time intervals at 37°C. Thereafter, a 20 μl MTT solution (5 mg/ml in PBS) was added to each well. After a 2 h incubation at 37°C, a 0.1 ml lysis buffer (20% SDS, 50% dimethylformamide) was added; incubation was performed for 1 h at 37°C, and the optical density (OD) at 570 nm was measured by Tecan plate reader.

### NF-κB DNA Binding Assay

Activation of NF-κB was evaluated by DNA binding assay using TransAM NF-κB Kit according to the manufacturer’s instructions and as previously described (Shanmugam et al., 2011b). Briefly, 20 μg of nuclear proteins extracted from NPO treated cells were added into 96-well plate coated with an unlabeled oligonucleotide containing the consensus binding site for NF-κB (5’-GGGACTTTCC-3’) and incubated for 1 h. The wells were washed and incubated with antibodies against NF-κB p65 subunit. An HRP conjugated secondary antibody was then applied to detect the bound primary antibody and provided the basis for colorimetric quantification.

### NF-κB Luciferase Reporter Assay

The effect of NPO on constitutive activation of NF-κB-dependent luciferase gene expression in HCC cells were determined as previously described (Shanmugam et al., 2011a). NF-κB responsive elements linked to a luciferase reporter gene were transfected with wild-type or dominant-negative IκB. The transfected cells were then treated with NPO for different time points up to 24 h. Luciferase activity was measured with a Tecan (Durham, NC, United States) plate reader and normalized to β-galactosidase activity. All luciferase experiments were done in triplicate and repeated twice.

### Flow Cytometric Analysis

To determine the effect of NPO on cell cycle distribution, HCCLM3 cells were treated with 10, 25 or 50 μM NPO for 48 h. At the end of treatment period the cells were collected, washed and fixed in ice cold 70% ethanol. The fixed cells were then washed with 1x PBS, resuspended with 0.1% RNase A in 1× PBS and stained with propidium iodide (25 μg/ml) [Sigma Aldrich, United States] for 30 min at room temperature. The apoptotic Sub-G1 cell population was determined using a CyAn ADP flow cytometer (Dako Cytomation).

### Western Blot Analysis

Nuclear extracts of NPO treated or control cells were prepared using TransAM nuclear extract kit according to the manufacturer’s instructions and as previously described (Shanmugam et al., 2011b). Briefly, 50 μg of nuclear proteins extracted from NPO treated or control cells was resolved in 10% SDS gel. After electrophoresis the proteins were electro-transferred to a nitrocellulose membrane (Bio-Rad, United States), blocked with Blocking One (Nacalai Tesque, Inc., Japan), and probed with p65, pp65 and Lamin B primary antibody overnight at 4°C. The blot was washed, and probed with horseradish peroxidase conjugated secondary antibody for 1 h and finally examined by chemiluminescence substrate (ECL, GE Healthcare, United Kingdom).

### Molecular Docking Analysis

To perform molecular docking, we retrieved the crystal structure of the heterodimer complex of IκBα/NF-κB (Huxford et al., 1998) from RCSB and used as a base our molecular docking studies. Using the CDOCKER protocol of Discovery Studio of Accelrys Product, we performed the molecular docking analysis to the heterodimer complex of IκBα/NF-κB by defining all residues in p65 within a radius of 4.5Å of any p50 atoms as binding site (Figure 5). Further, we carried out a molecular docking run using DS default parameters. Resulting protein-ligand complexes were visualized using the DS visualization tool, and the analysis was tabulated.

### Acute Toxicity Studies

All animal experiments were performed according to protocols approved by the Sing Health Institutional Animal Use and Care Committee. For acute toxicity study, eight week-old NCr nude Female mice (In vivos, Singapore) were treated with intra-peritoneal injections of 5 mg/kg, 25 mg/kg, 50 mg/kg, of NPO and vehicle (0.1% DMSO). The mice were monitored daily, for development of any toxic signs, including change in physical appearance, hunched back, increased respiration, arching and rolling, muscle spasm, tremors, cyanosis, stimulation or depression. The changes in body weight, food and water intakes were monitored daily for up to day 8. On day 8, cardiac puncture was employed for terminal blood collection from the mice. The liver and kidney functions were determined using the serum collected. ALT (SGPT) as well as AST (SGOT) were used to assess liver functions and BUN was used to evaluate kidney function as described previously (Dai et al., 2017).

### Orthotopic implantation of HCC in SCID Mice

HCCLM3 cells were maintained in Dulbecco’s modified Eagle’s medium (DMEM) containing 10% fetal bovine. HCCLM3-luciferase (HCCLM3-Luc) cells were generated by transfecting HCCLM3 cells with the firefly luciferase (FLUC) gene. 100 μl medium containing 3 × 10^6^ HCCLM3-Luc cells were injected subcutaneous in the right flank of nude mice. When the tumor volume reached approximately 1 cm^3^, tumor was harvested, cut into 2 mm^3^ pieces and implanted orthotopically into the liver of Female NCr nude mice. A midline abdominal incision (3–5 cm) was made to expose the whole liver and the liver capsule was mechanically injured with a needle. Then a piece of HCC tissue (about 2 mm^3^) was filled into the liver of the recipient mouse with forceps and the abdominal wall closed. The skin incisions were closed with wound clips. The development of tumors was monitored by measuring the bioluminescence signals and was quantified using the Xenogen IVIS system (Caliper Life Sciences, CA, United States) as described previously (Dai et al., 2017).

### Orthotopic HCC Tumor Model

All animal experiments were performed according to protocols approved by the SingHealth Institutional Animal Use and Care Committee. For drug efficacy study, eight week-NCr nude Female mice (Invivos, Singapore) were implanted orthotopically with HCCLM3-Luc cells-induced tumors. When the bioluminescence signal reaches 10^6^, mice were grouped into 2 groups and treated with vehicle (0.1% DMSO) and 50 mg/kg of NPO, twice a week by intra-peritoneal injections for 4 consecutive weeks. The development of tumor was monitored twice a week by measuring the bioluminescence signals. Mice were euthanized when the humane end-point criteria is met by CO_2_ inhalation. Primary tumor (liver) and lung tissues were excised, snap-frozen and stored at –80°C until used for experiments.

### Statistical Analysis

Student *t*-test was used to analyze the data. For *in vivo* studies, unpaired *t*-test with Welch’s correction was used for statistical comparisons between groups. *p* < 0.05 was considered statistically significant (GraphPad Prism 5.0; GraphPad Software, CA, United States).

## Results

### Chemistry

**FIGURE 1:**
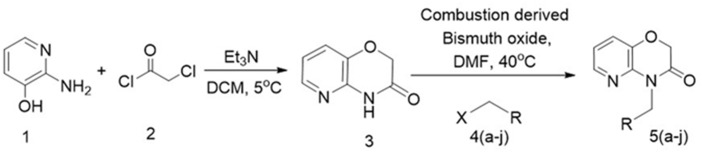
Schematic representation for the synthesis of N-substituted pyrido1,4-oxazin-3-ones.

In the present report, we report the synthesis of N-substituted pyrido-1,4-oxazin-3-ones. Initially, 2-aminopyridine-3-ol (**1**) was allowed to react with chloro acetylchloride (**2**) at 5°C in basic medium to yield 2H-pyrido[3,2-b][1,4]oxazin-3(4H)-one (**3**). Next, we prepared N-substituted pyrido1,4-oxazin-3-ones (**5a-j**, Table 1) by reacting **3** with various alkyl bromides (**4a-j**) in the presence of combustion derived bismuth oxide in DMF solvent (Figure 1). The compounds obtained were characterized by melting point, ^1^H NMR, ^13^C NMR, elemental and mass spectral analysis. Detailed chemical characterizations of the newly synthesized compounds are provided in experimental section and chromatograms are provided in Supplementary File.

**Table 1:** Library of synthesized N-substituted pyrido-1,4-oxazin-3-ones.

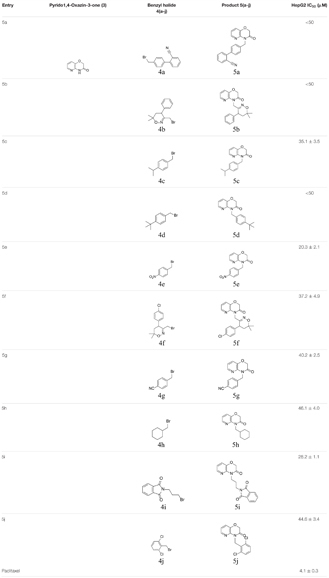

### Pharmacology

#### Growth Inhibitory Activity of N-Substituted Pyrido-1,4-Oxazin-3-Ones Against HCC Cells

**FIGURE 2:**
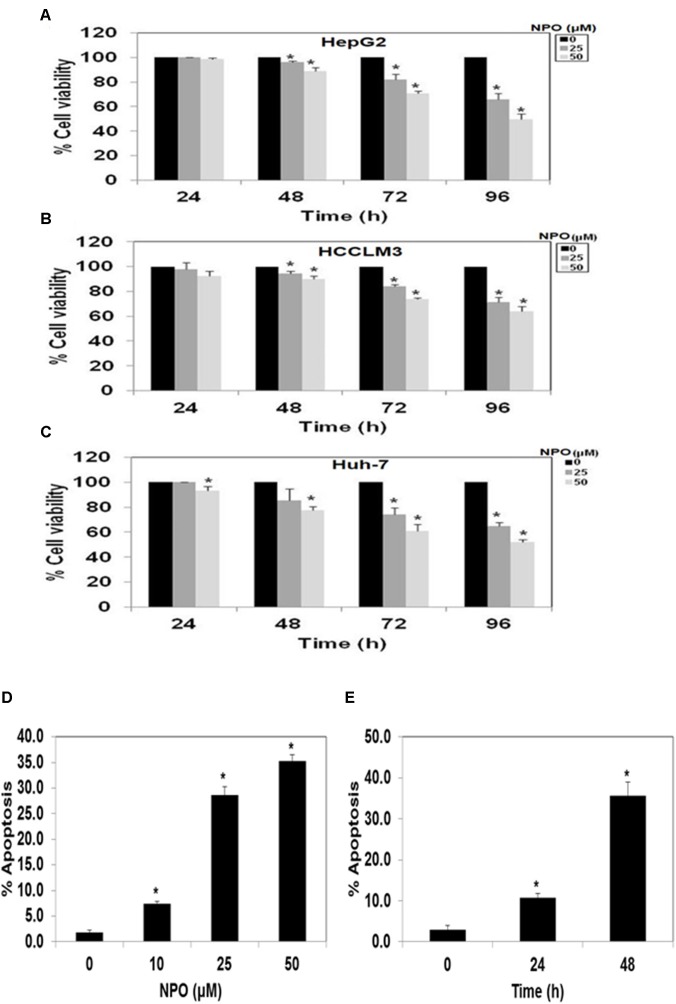
**(A–C)** NPO the lead compound significantly inhibits the viability of various HCC cells lines. NPO was identified as lead anticancer agent among the structural variants and NPO decreased the growth of HepG2, HCCLM3 and Huh-7 cells in dose- and time-dependent manner. **(D)** and **(E)**, NPO can induce apoptosis in a dose and time-dependent manner in HCCLM3 cells. ^∗^*p* < 0.05.

The library of synthesized N-substituted pyrido-1,4-oxazin-3-ones were tested for their growth inhibitory activity against HepG2 cells using MTT assay, the results are summarized in Table 1. Among the tested compounds, the compound 5e (NPO) significantly inhibited the proliferative activity of HepG2 cells, with an IC_50_ values of 20.3 μM. The presence of nitro group in phenolic ring was found to be potent antiproliferative agent against HepG2 cells. The N-substituted pyrido 1,4-oxazines bearing chlorine atom and nitrile group were not notably effective and the incorporation of methyl groups and isopropyl group to phenyl ring diminishes its potency as noted regarding the compounds 5c and 5d. Further, introduction of isoindoline-1,3-dione group (5i) on pyrido1,4-oxazine resulted a moderately enhanced inhibition with an IC_50_ value of 28.2 μM. We next examined the effect of NPO on the viability of panel of HCC (HepG2, HCCLM3, and Huh-7) cells in different dose and time points (Figure 2). We observed significant reduction in the viability cells on treatment with NPO in time- and dose-dependent manner.

#### NPO Abrogates Constitutive Activation of NF-κB in HCC Cells

**FIGURE 3:**
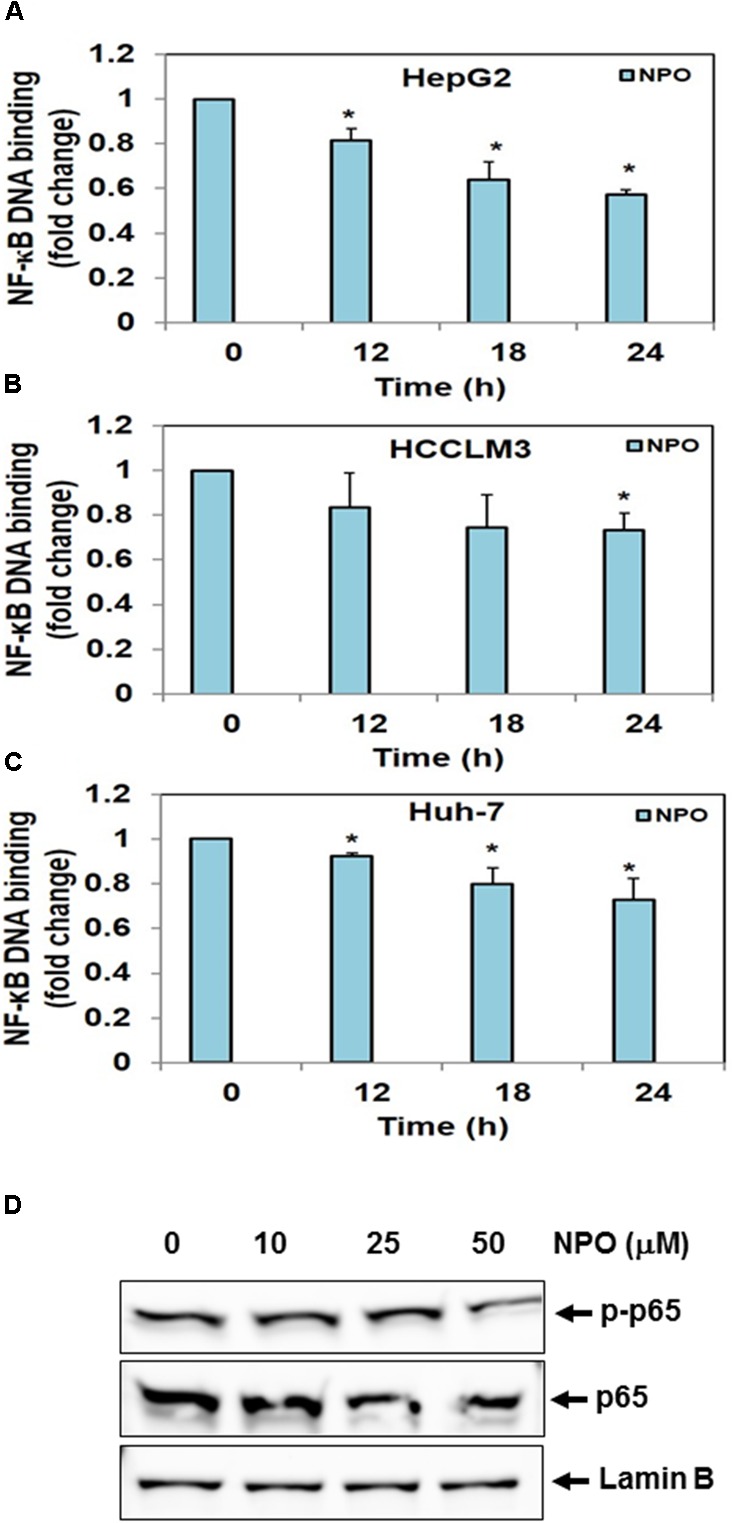
**(A–C)** NPO downregulates NF-κB DNA binding ability in HCC cells. HepG2, HCCLM3, and Huh-7 cells were treated with NPO for different time points, nuclear extracts were prepared, and 20 μg of nuclear extract protein was used for DNA binding assay. The NF-κB DNA binding was inhibited in time-dependent manner. **(D)** Effect of NPO on p65 phosphorylation in HCCLM3 cells. Nuclear extracts were prepared as described in Materials and Methods. HCCLM3 cells were treated with NPO at doses of 10, 25, and 50 μM for 24 h and expression of various proteins was analyzed by western blot analysis. ^∗^*p* < 0.05.

Oh and coworkers performed high-throughput screening of most diverse 7,243 compounds using time-resolved fluorescence resonance energy transfer and identified three scaffolds namely 7-benzoyl-4-phenylcyclopenta[1,2] oxazine, 1-(thiophen or furan)-2,3-dihydroimidazo[1,5] pyridine and 2-phenyloxazolo[5,4] pyridine as potent IKKβ inhibitors, and in turn disrupts NF-κB signaling pathway (Oh et al., 2010). Therefore, we evaluated the effect of lead pyrido-oxazinone, NPO on constitutive activation of NF-κB in HepG2, HCCLM3, and Huh-7 cells. The treatment of HCC cells with NPO at different time points (0, 12, 18, and 24 h) suppressed the persistent activation of NF-κB in HCC cells in a time-dependent manner (Figure 3).

#### NPO Induces Increased Accumulation of Cells in the Sub-G1 Phase of the Cell Cycle

Results from the flow cytometry analysis clearly indicated that the NPO induces accumulation of cells in the sub-G1 phase which is indicative of apoptosis in a dose and time-dependent manner (Figures 2D,E). Taken together, these data suggest that NPO is a potent inducer of apoptosis in HCC cells.

#### NPO Inhibits NF-κB Dependent Luciferase Expression in HCC Cells

**FIGURE 4:**
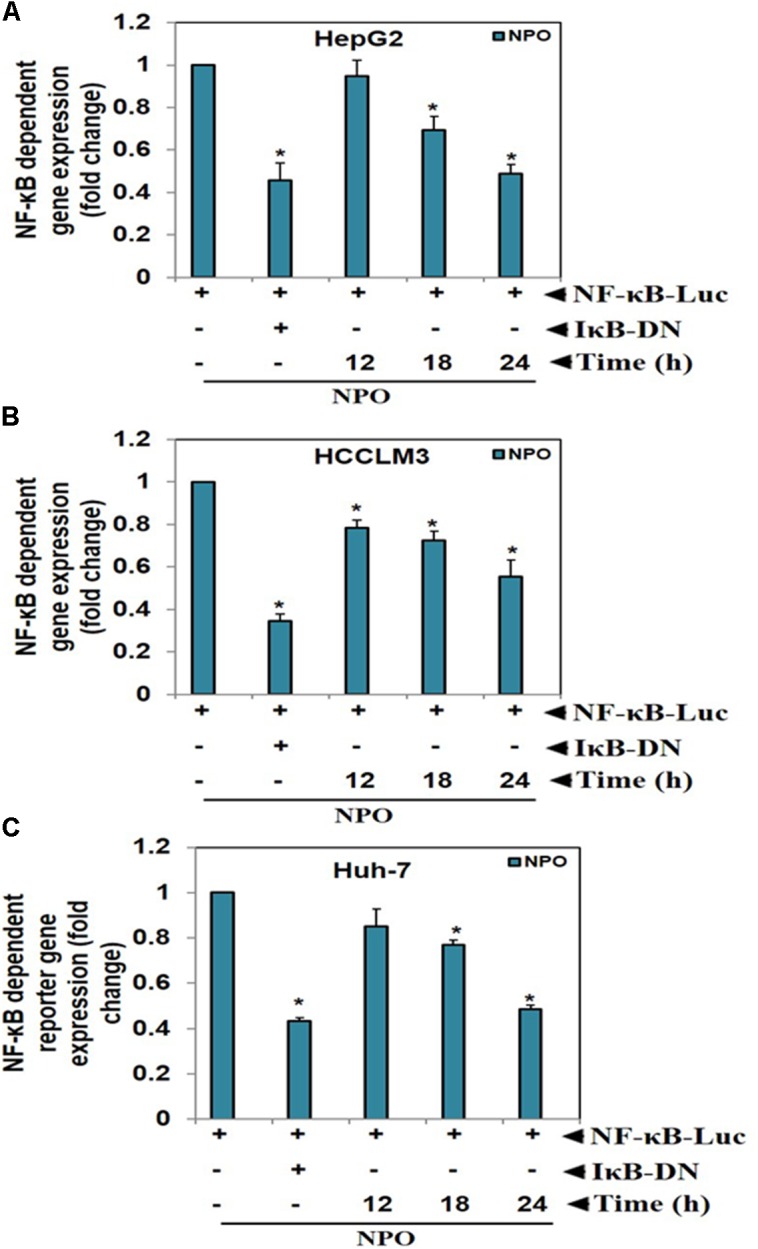
NPO inhibits NF-κB dependent luciferase expression in HCC cells: with NF-κB responsive elements linked to a luciferase gene were transfected with wild-type or dominant-negative IκB and transfected **(A)** HepG2, **(B)** HCCLM3, and **(C)** Huh-7 cells were treated with NPO at indicated time points and reporter (luciferase) activity was measured. All luciferase experiments were done in triplicate and repeated twice (^∗^*p* < 0.05).

Transcription is a multifactorial process and suppression of DNA binding activity of NF-κB alone does not always correspond to the inhibition of its transcriptional activity. Therefore, we transfected HepG2, HCCLM3, and Huh-7 cells as described in methods and evaluated the effect of NPO on the NF-κB dependent luciferase expression. The treatment of HCC cells with NPO reduced NF-κB regulated reporter gene expression in a time-dependent manner with the maximum inhibition of nearly 50% (Figure 4). These results demonstrate that NPO reduces DNA binding ability of NF-κB and in turn downregulates NF-κB regulated gene expression.

#### NPO Decreases p65 Phosphorylation in HCC Cells

We next investigated the effect of NPO on p65 phosphorylation in HCC cells. The data reveals that NPO can indeed abrogate p65 phosphorylation in a dose dependent manner in HCCLM3 cells (Figure 3D).

#### *In silico* Interaction of NPO With NF-κB

**FIGURE 5:**
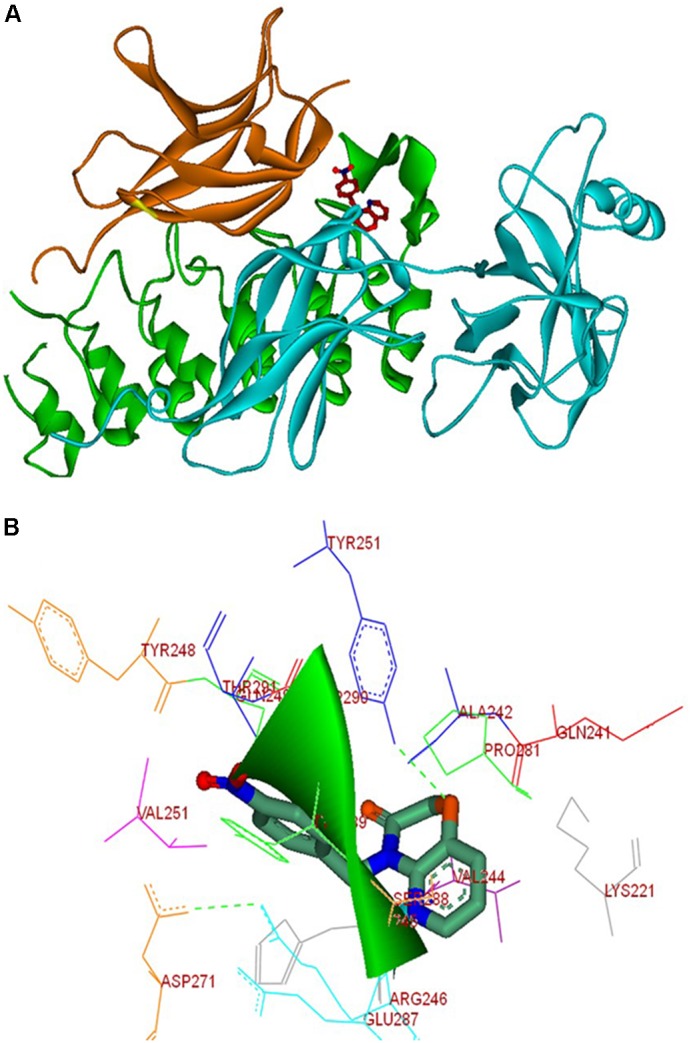
Molecular interactions between NPO and the IκBα/NF-κB complex. **(A)** Molecular docking solution for NPO (in stick representation, and atomic coloring with orange) targeting IκBα/NF-κB complex. The p50 sub-unit is show in orange, p65 in cyan, and IκBα in green; **(B)** the interaction map of the complex formed between NPO and IκBα/NF-κB complex is shown.

In order to understand the molecular interaction of NPO within the heterodimeric complex of IκBα/NF-κB, the molecular docking simulations of the reported compounds were performed using CDOCKER program (Wu et al., 2003). We used the crystal structure of heterodimer of IκBα and NF-κB (PDB ID: 1IKN). Using the CDOCKER protocol of Accelrys DS Version 2.5, we performed the molecular docking studies. All the compounds were well docked into the interface of the heterodimeric complex of NF-κB. Analysis of the results shows that NPO orient into the p65 interaction region of IκBα, which comprises of amino acids like Gln249, Tyr248, Glu287, Ser288, Tyr289, Asp290, Thr291, and Pro281 with a variety of interactions (Figure 5). Further, the condensed pyrido-1,4-oxazin-3-one ring of NPO buried into the hydrophobic pocket made up of Val244, Ala242, His245, and Lys221 of the p65 subunit of NF-κB. The ring was presented to interact with Arg246 of P65 subunit and Tyr251 of IκBα on the other side via hydrogen bonding indicates that preferential binding of NPO to NF-κB complex.

#### NPO Treatment Did Not Induce Adverse Effects *in vivo*

**FIGURE 6:**
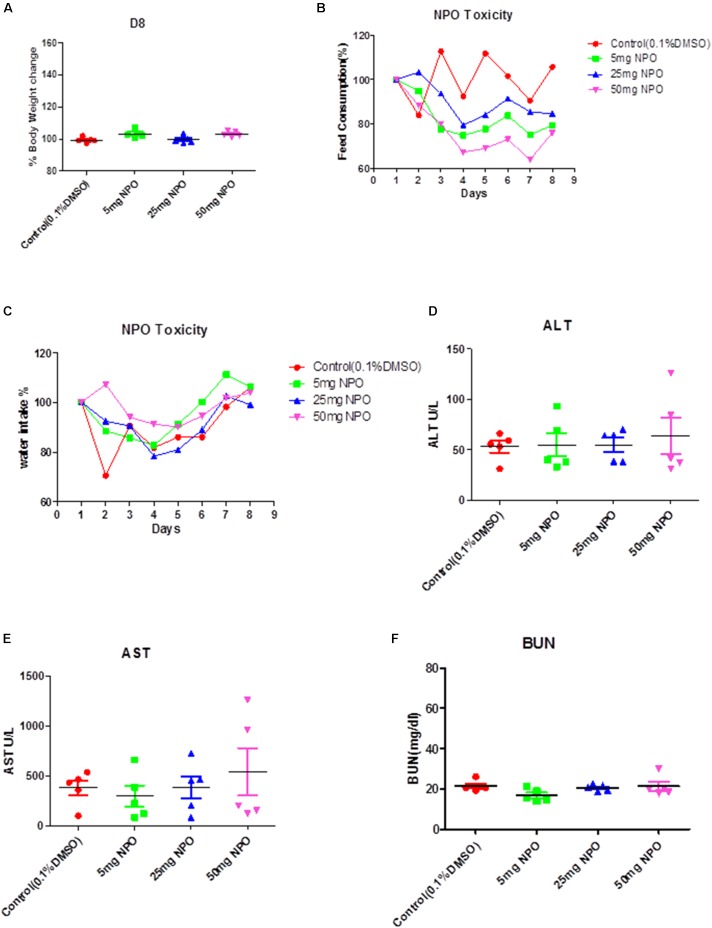
Acute toxicity studies with NPO. **(A)** The effect of intraperitoneal administration of NPO on body weight change. The nude mice were treated with one single dose of NPO (5, 25, or 50 mg/kg). Error bars are means ± SD. Ordinary one-way ANOVA. **(B,C)** The effect of NPO on mice behavior study including feed consumption **(B)** and water intake **(C)**. Error bars are means ± SD. Ordinary one-way ANOVA. **(D–F)** Effect of NPO on biochemical parameters such as ALT, AST, and BUN levels. Error bars are means ± SD. Ordinary one-way ANOVA.

We initially performed acute toxicity studies with NPO to determine its sub-lethal dose for *in vivo* studies. The mice were monitored for 8 days after the intraperitoneal administration of the 5, 25, or 50 mg/kg dose of NPO and vehicle (0.1% DMSO). At the end of the experiment, no mortality was observed, which indicated that the LD_50_ must be higher than 50 mg/kg. As indicated in the Figures 6A–C, there was no significant difference of behavioral and physical symptoms such as body weight, feed consumption, and water intake between NPO treated and the control group. The various biochemical parameters of the serum including alanine aminotransferase (ALT), aspartate aminotransferase (AST), and blood urea nitrogen (BUN) were detected by the autoanalyzer. It was noted that compared to the control group, no substantial differences were found in the serum levels of ALT, AST, and BUN in the NPO-treated groups. All these results indicate that the intraperitoneal administration of NPO did not produce any obvious toxic effects in NCr nude mice (Figures 6D–F).

#### NPO Inhibited Tumor Growth in an Orthotopic HCC Mouse Model

**FIGURE 7:**
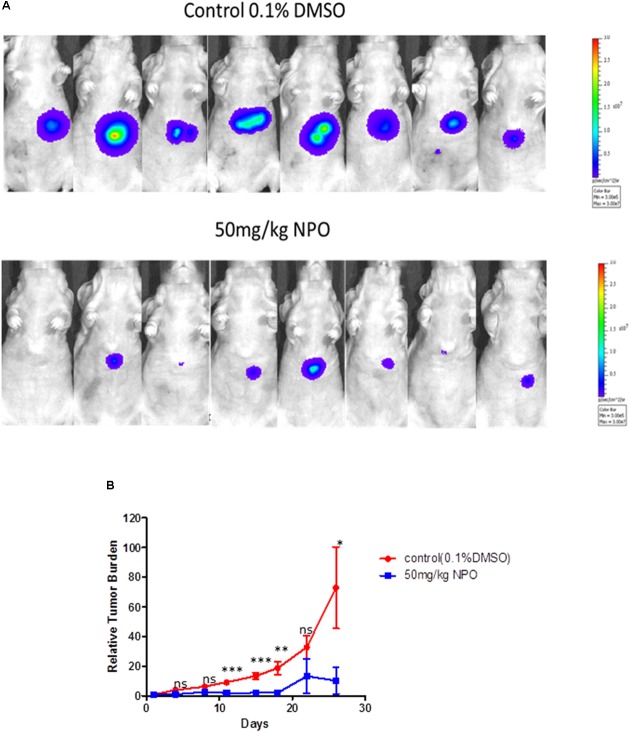
NPO significantly abrogates tumor growth in an orthotopic mouse model. **(A)** Bioluminescence images of orthotopic tumor bearing mice. Ncr nude mice were orthotopically implanted with HCCLM3_Luc cells and then treated with 0.1% DMSO (*n* = 8) or 50 mg/kg NPO (*n* = 8) for 26 days. **(B)** The scatter plot represents the differences in tumor burden between control and NPO treated mice and is expressed as tumor burden relative to photon counts. The statistics was determined using unpaired *t* test with Welch’s correction. ^∗^*p* < 0.05; ^∗∗^*p* < 0.01; ^∗∗∗^*p* < 0.001.

We also analyzed the anti-tumor potential of NPO *in vivo* through intraperitoneal administration using the HCCLM3_Luc orthotopic model. After treatment with 50 mg/kg of NPO (three doses per week for 4 consecutive weeks), bioluminescence images revealed that there was a significant reduction of tumor growth in the NPO group compared with the vehicle control group at the end of the assay (Figure 7A). The differences in tumor burden at the last time point was quantitated by measuring photon counts and expressed as the tumor burden relative to photon counts before the first therapeutic injection. An unpaired *t* test with Welch’s correction indicated that the NPO treatment group had significant inhibition of tumor burden compared with the vehicle-treated controls (Figure 7B).

## Discussion and Conclusion

NF-κB has been reported to be persistently activated in several types of cancer including solid (breast and prostate) tumors and hematopoietic (multiple myeloma, chronic myelogenous leukemia, acute myelogenous leukemia, acute lymphocyte leukemia) tumors which render it as an important molecular therapeutic target in designing novel anti-cancer therapeutics (Nakshatri et al., 1997; Feinman et al., 1999; Palayoor et al., 1999; Kordes et al., 2000; Griffin, 2001; Aggarwal, 2004). NF-κB regulates the expression of various oncogenic genes and plays a crucial role in regulation of inflammation, stress response and immune system. Dysregulation of NF-κB signaling can promote neoplastic transformation, cancer progression and tumor evasion (Plati et al., 2008). Hence researchers have rightly labeled the activation of NF-κB as the enemy within the cell (Aggarwal).

Prior studies have also established the presence of NF-κB proteins in advanced ovarian cancers and its association with poor overall survival (Annunziata et al., 2010). Analysis of NF-κB in clear cell renal cell carcinoma tissues revealed the expression of NF-κB in 85% of patient tissues and thereby presented NF-κB as a potential therapeutic target in the treatment of clear cell renal cell carcinoma (Meteoglu et al., 2008). Li and coworkers reported the possible involvement of IL-17A induced NF-κB activation in metastasis and poor prognosis of HCC (Li et al., 2011). In the present study, we have prepared a novel class of pyrido-1,4-oxazin-3-ones and identified the lead anticancer structure against HCC cells.

This study introduces a novel N-alkylation procedure to prepare N-substituted pyrido-1,4-oxazin-3-ones. Functional studies further suggested that lead compound can interfere with DNA binding ability of NF-κB and may inhibit relay of downstream signaling and in turn can modulate the expression of NF-κB regulated genes.

Additionally, the preclinical safety and efficacy of NPO was further confirmed using nude mice. It was noted that no obvious toxicity was observed after treatment with NPO at 5 mg/kg, 25 mg/kg and 50 mg/kg by intraperitoneal injections. Interestingly, we also found that NPO at a dose of 50 mg/kg could significantly attenuate tumor growth in an orthotopic mouse model. Overall, these findings establish the potential role of NPO as a novel NF-κB blocker that can find suitable application for the treatment of diverse malignancies.

## Author Contributions

KR, B, GS, APK, PL, TA, and AB conceived the project. KR, B, D, GS, and CM designed the experiments. HB, B, SR, MS, AC, SA, and AD carried out the research and analysis of data. KR, B, GS, APK, CM, HB, and KH wrote the paper.

## Conflict of Interest Statement

The authors declare that the research was conducted in the absence of any commercial or financial relationships that could be construed as a potential conflict of interest.

## Supplementary Material

DATA SHEET S1 Effect of NPO on p65 and pp65 phosphorylation in HCCLM3 cells. Nuclear extracts were prepared as described in Materials and Methods. HCCLM3 cells were treated with NPO at doses of 10, 25, and 50 mM and expression of various proteins was analyzed by western blot analysis.

DATA SHEET S2 NPO can induce apoptosis in a dose dependent manner in HCCLM3 cells.

DATA SHEET S3 NPO can induce apoptosis in a time-dependent manner in HCCLM3 cells.
