# Supplementary figures and images for: N-Substituted Pyrido-1,4-Oxazin-3-Ones Induce Apoptosis of Hepatocellular Carcinoma Cells by Targeting NF-κB Signaling Pathway
Source: Front Pharmacol. 2018 Nov 5;9:1125. doi: 10.3389/fphar.2018.01125 (PMC6230568; doi:10.3389/fphar.2018.01125)

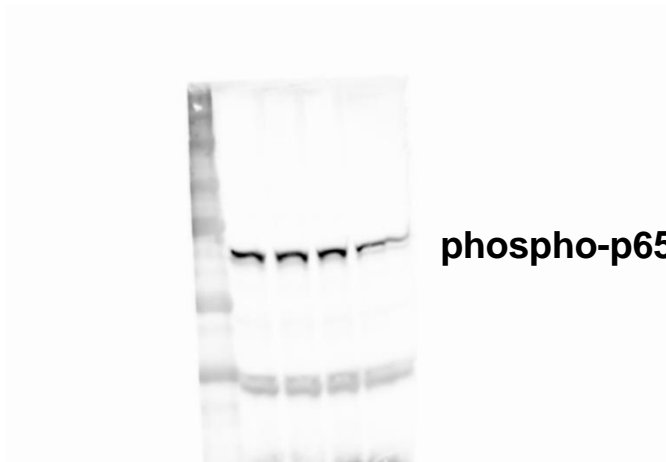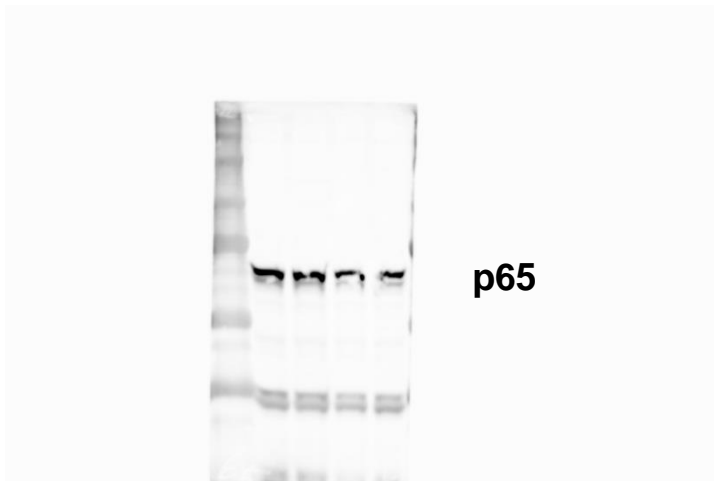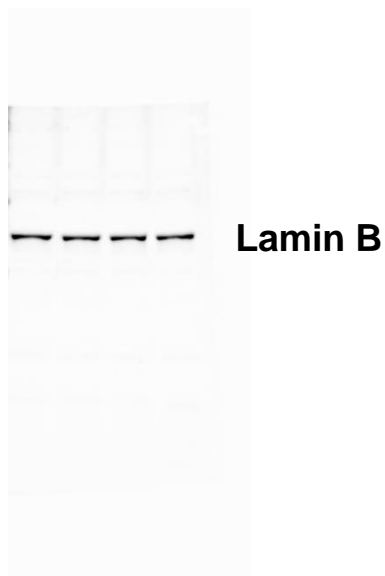

Supplement: DATA SHEET S1 — Effect of NPO on p65 and pp65 phosphorylation in HCCLM3 cells. Nuclear extracts were prepared as described in Materials and Methods. HCCLM3 cells were treated with NPO at doses of 10, 25, and 50 mM and expression of various proteins was analyzed by western blot analysis. [file Data_Sheet_1.PDF]

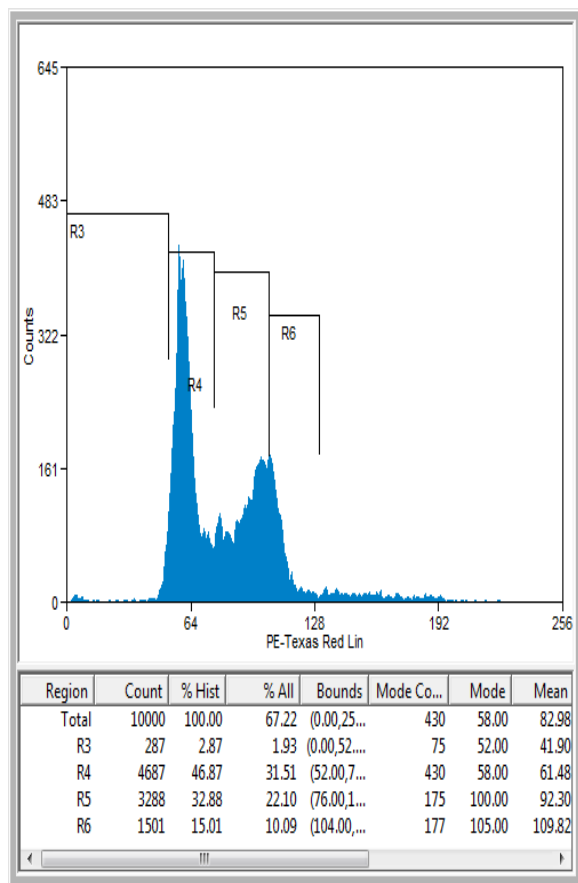

Control 1

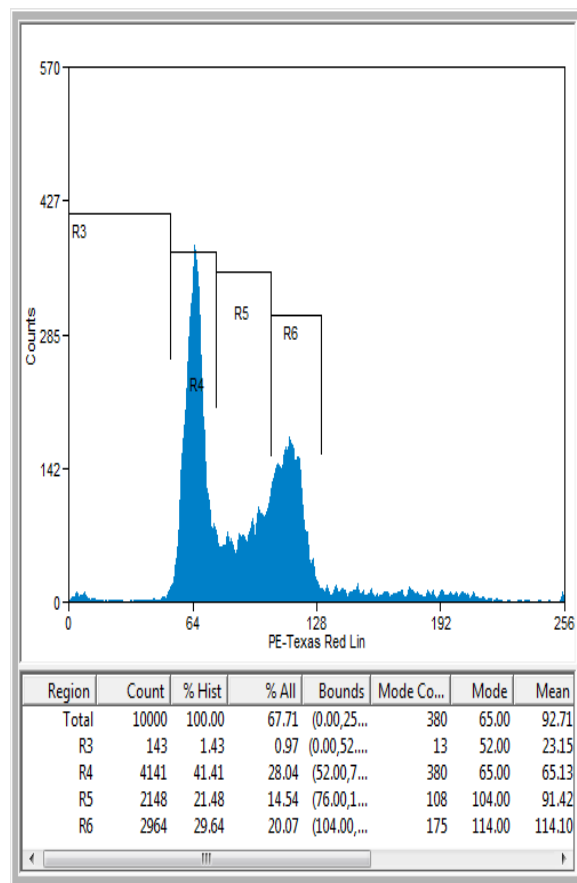

Control 2

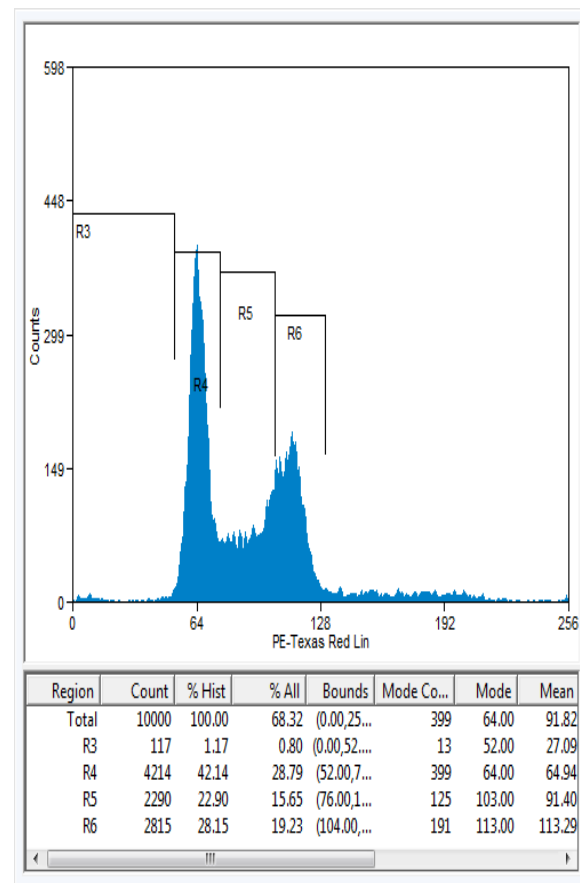

Control 3

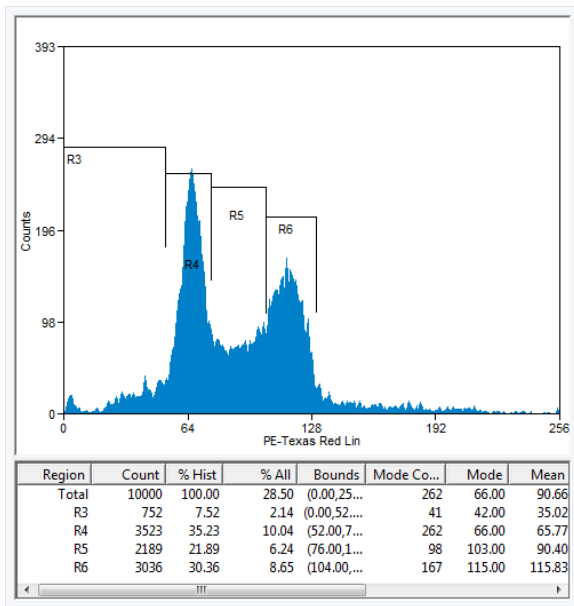

10 uM- 1

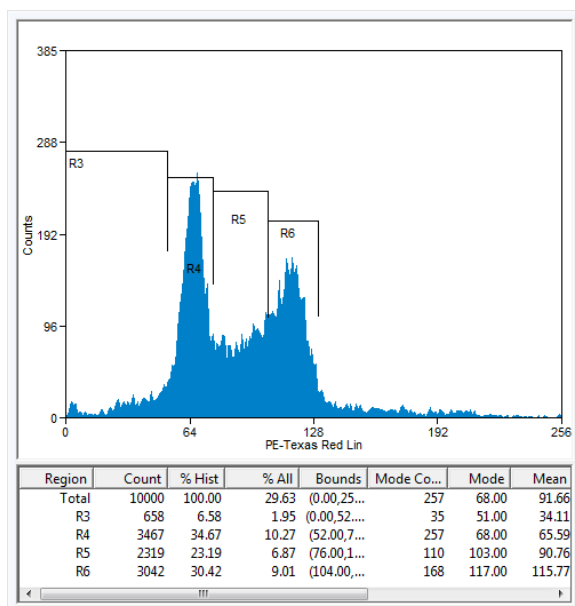

10 uM- 2

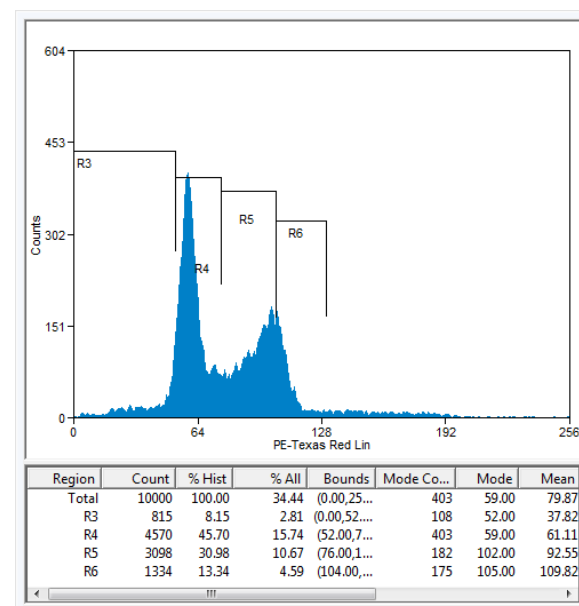

10 uM- 3

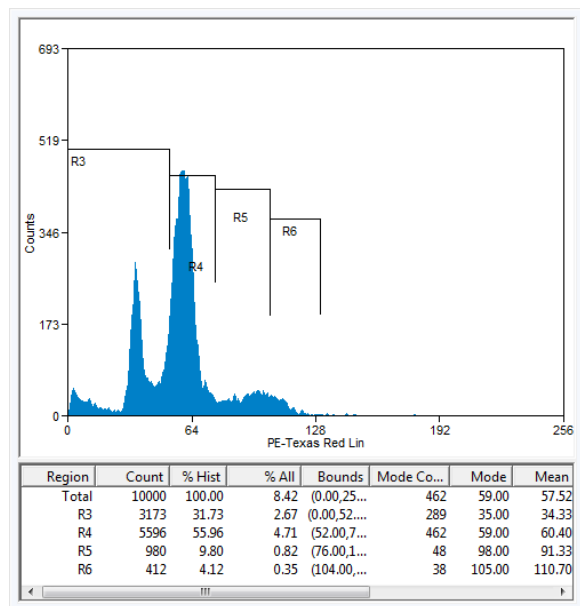

25 uM- 1

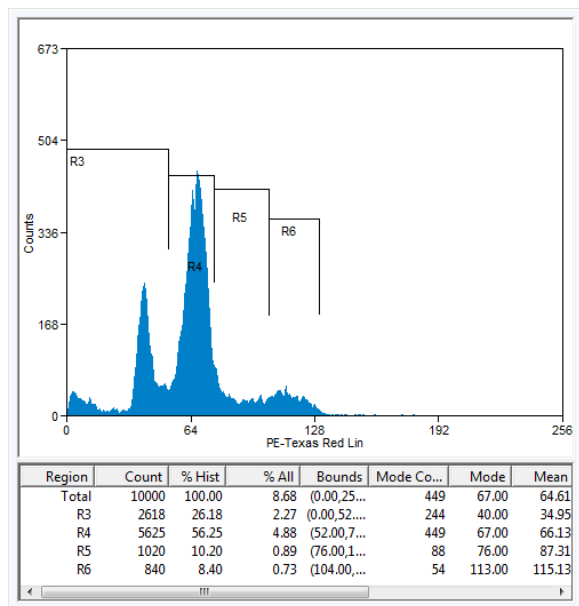

25 uM- 2

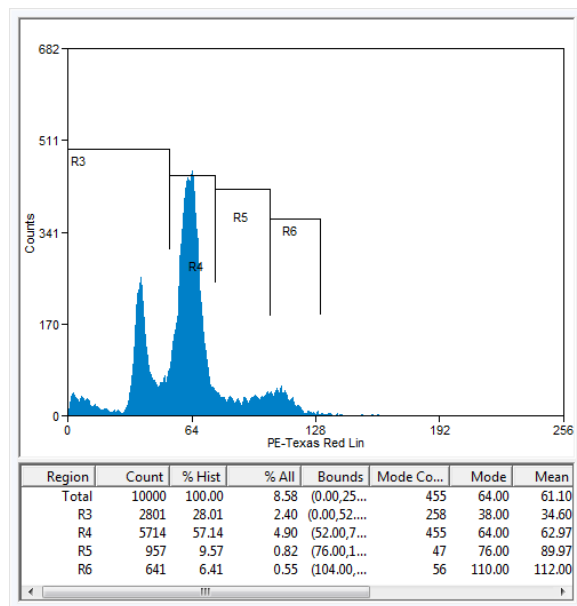

25 uM- 3

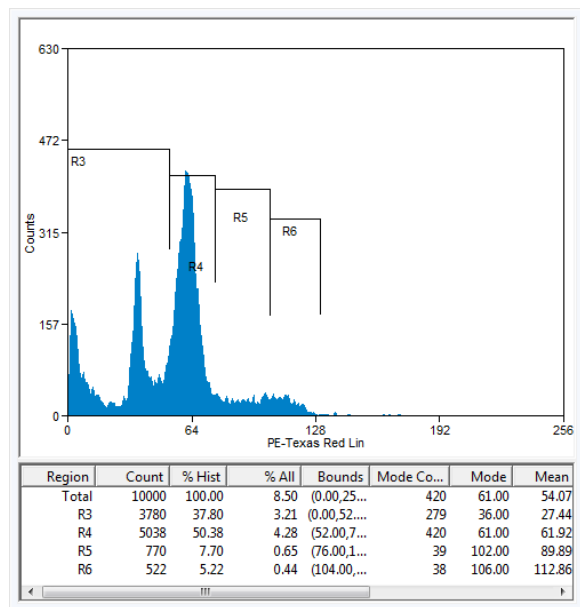

50 uM- 1

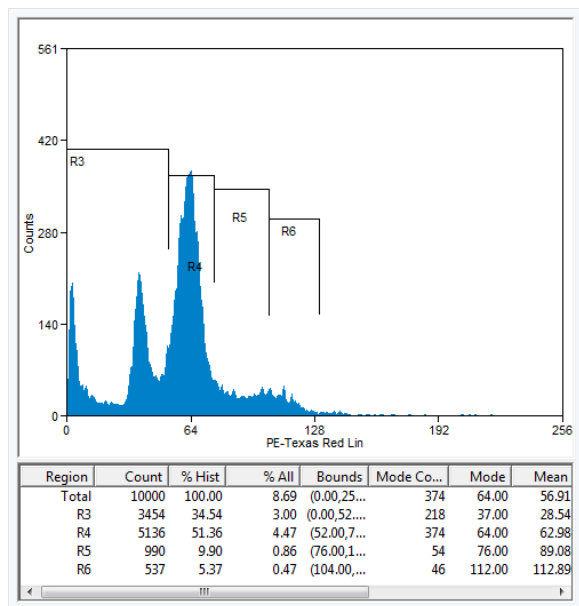

50 uM- 2

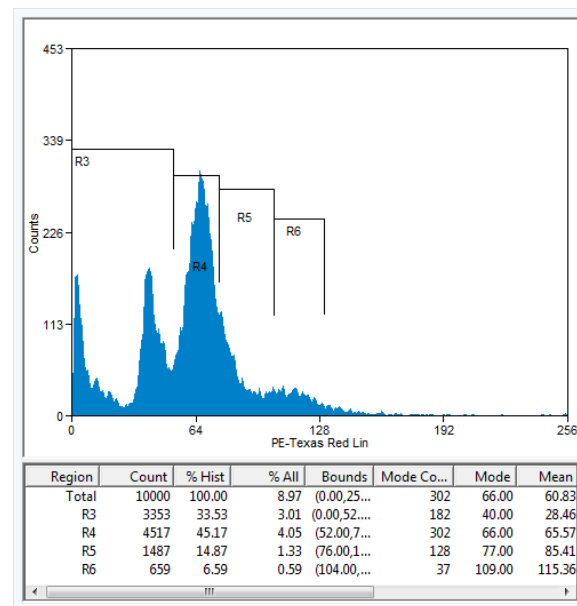

50 uM- 3

Supplement: DATA SHEET S2 — NPO can induce apoptosis in a dose dependent manner in HCCLM3 cells. [file Data_Sheet_2.pdf]

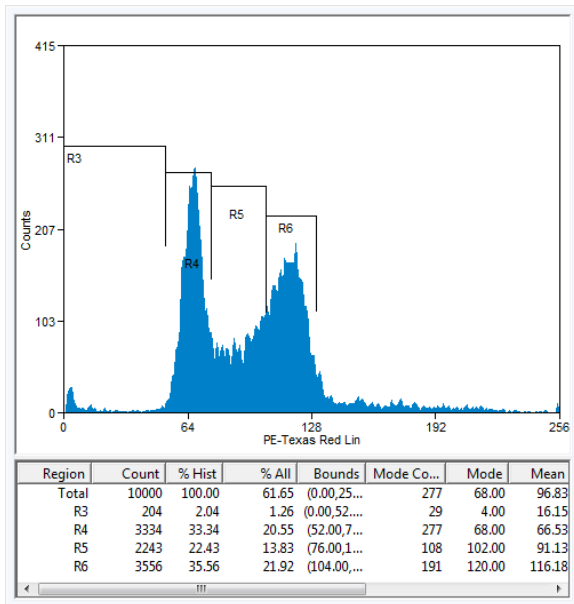

Control 4

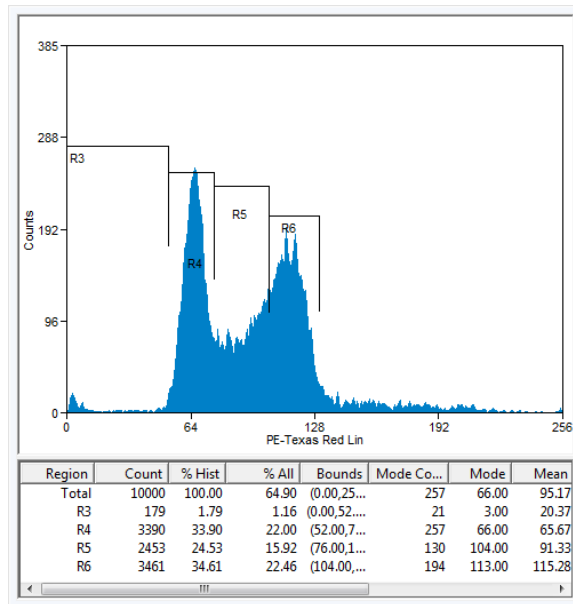

Control 5

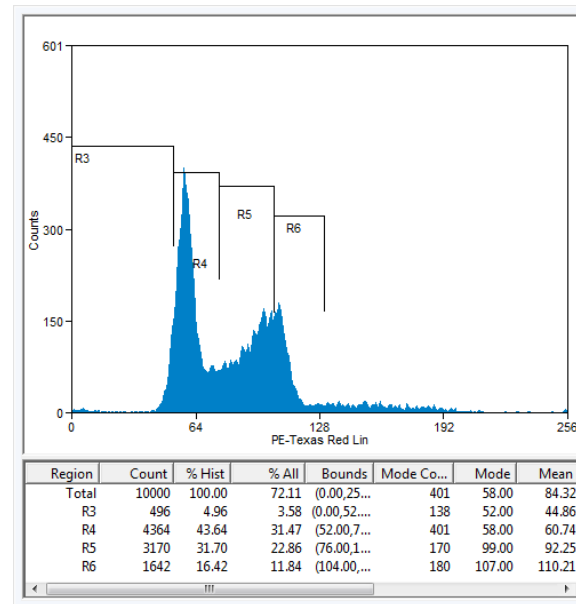

Control 6

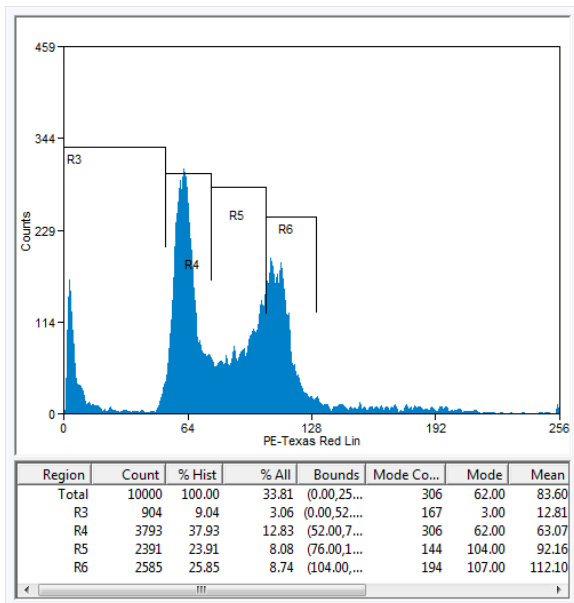

24 h- 1

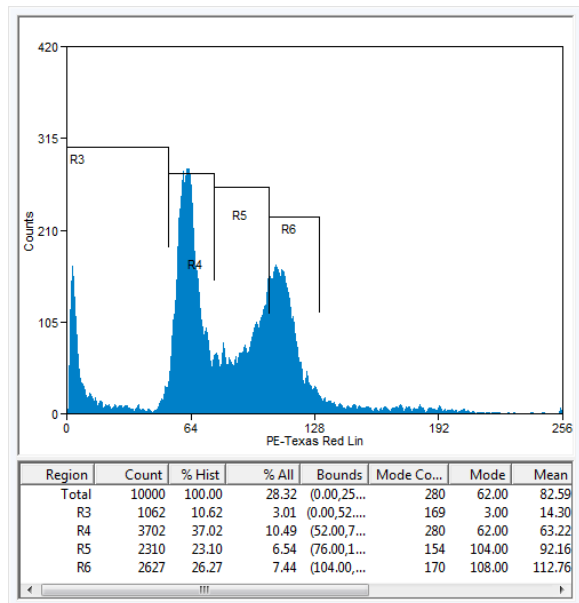

24 h- 2

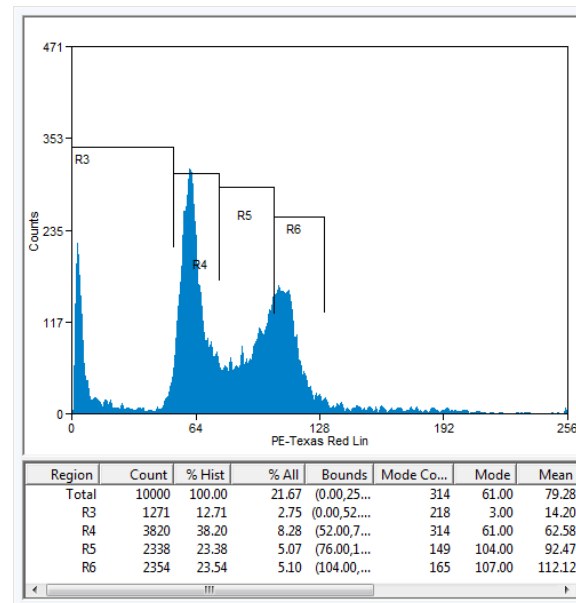

24 h- 3

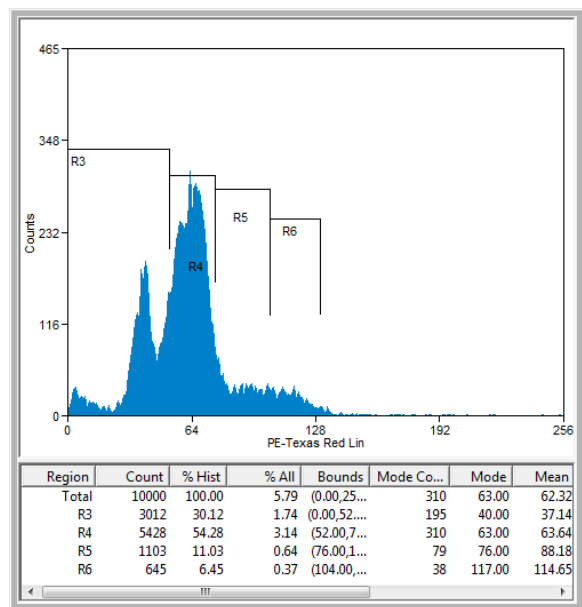

48- 1

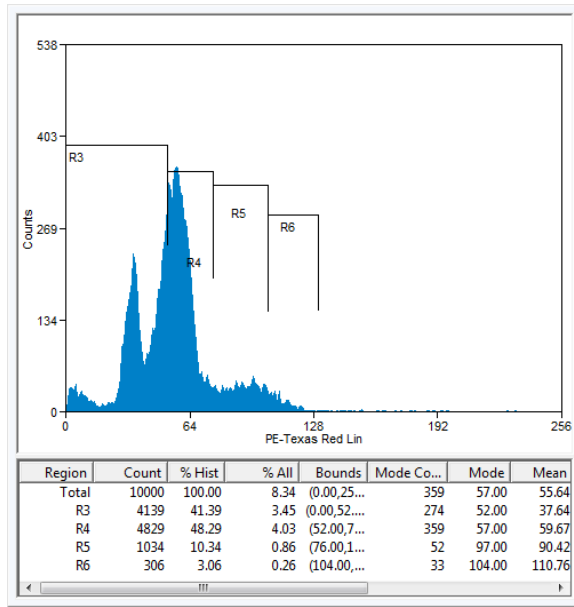

48- 2

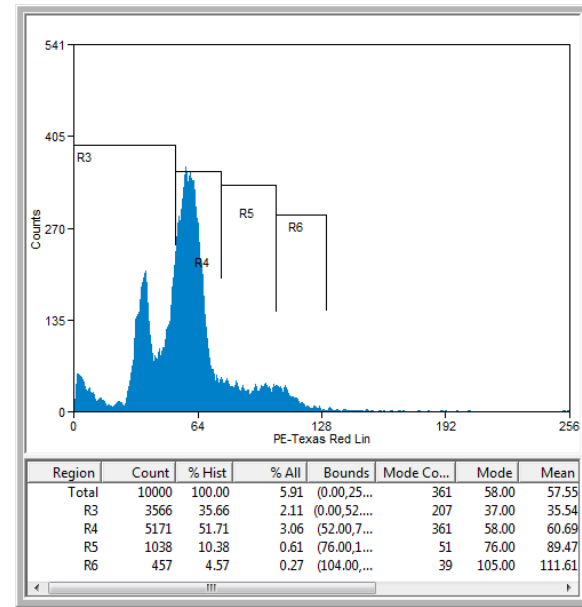

48- 3

Supplement: DATA SHEET S3 — NPO can induce apoptosis in a time-dependent manner in HCCLM3 cells. [file Data_Sheet_3.pdf]
